# Supplementary material for: Allied Health Professions Accreditation Standards for Work Integrated Learning: A Document Analysis
Source: Int J Environ Res Public Health. 2023 Jul 31;20(15):6478. doi: 10.3390/ijerph20156478 (PMC10418477; doi:10.3390/ijerph20156478)
Supplement: Supplementary file 1 [file ijerph-20-06478-s001.zip › ijerph-2453814-supplementary.pdf]

Supplementary Table S1. Work integrated learning and placements across the professions.

(NB: Italicised text represents text extracted exactly as presented in their respective source documents.)

Allied Health Profession	How accreditation documents describe the purpose/priorities/emphasis of what work integrated learning is for the profession	Quantity/Volume of placements	Supervision requirements for placements
Chinese Medicine	Safe practice is emphasised and expected to be included in learning outcomes of any work-integrated learning components.<br>Students are expected to achieve relevant pre-clinical capabilities before each work-integrated learning experience.<br>Work-integrated learning provides opportunity for integration of theory and practice.<br>Work-integrated learning helps students to demonstrate achievement of the professional capabilities.<br>Provides an opportunity for students to reflect on observations of professional practice.	*The quality, quantity, duration and diversity of student experience during work-integrated learning in the program is sufficient to produce a graduate who has demonstrated the knowledge, skills and professional attributes to safely and competently practise Chinese medicine.* (Standard 3.12) [37] (p. 19)<br>Expectation that students participate in extensive and diverse experiences across a range of clients (e.g. different ages, cultural backgrounds) and clinical presentations (e.g. musculoskeletal issues, internal disorders).	Supervisors must hold current registration in Australia.<br>Supervision can be direct, indirect or remote and is dependent of the context.

Allied Health Profession	How accreditation documents describe the purpose/priorities/emphasis of what work integrated learning is for the profession	Quantity/Volume of placements	Supervision requirements for placements
Chiropractic	Safe practice is emphasised and expected to be in the guiding principles of clinical training student learning outcomes. Students are expected to achieve relevant competencies before providing patient care.	*The quality and quantity of clinical experience are sufficient for developing a student to be a graduate competent to practise.* (Standard 3.7) [37] (p. 19)	Supervisors must be registered, suitably qualified & experienced chiropractors and/or health practitioners
Dietetics	Workplace learning experiences provide students with opportunities to develop and demonstrate professional competence across a range of settings and population groups. Students are prepared for placement.	Supervised workplace learning experiences that support students to meet the national competency standards. (Standard 4.1) [42] (p. 9)<br>A minimum of 100 equivalent working days across the program. (Standard 4.1) [42] (p. 9)<br>There are a variety of workplace learning experiences *reflecting socio-ecological approaches to health, major health priorities and the broad landscape of dietetic practice, including policy and the provision of services and care to individuals, groups, communities and populations, which allows students to meet the national competency standards.* (Standard 4.2) [42] (p. 9)<br>Placements can occur across a variety of settings. There is no minimum time that	Supervisors must have current qualifications and experience relevant to the areas they are supervising.

Allied Health Profession	How accreditation documents describe the purpose/priorities/emphasis of what work integrated learning is for the profession	Quantity/Volume of placements	Supervision requirements for placements
		students are expected to be on placement in each setting. For placements within one setting, there must be a wide variety of experiences. Project-based placements are permitted.	
Exercise Physiology	Practicum is integrated into the course curriculum.<br>Content that prepares students in terms of developing knowledge and skills to be able to undertake a meaningful placement experience is completed before practicum.<br>Practicum provides students with opportunities students to apply their knowledge and skills and to demonstrate their competence in a real-world setting.<br>Practicum allows students to gain experience in inter-professional practice.	*A minimum of 360 hours in a variety of activities to demonstrate attainment of competency in exercise assessment and prescription and delivery. Of these hours:*<br>*- at least 200 hours across the Accredited Exercise Physiology (AEP) core areas of practice.*<br>*- remaining 160 hours can be in any area across the AEP scope of practice, but with no more than 100 hours across the emerging or niche areas of practice.*<br>(Practicum Standard 2.2.1) [48] (p. 3)<br>Practicum is undertaken across a range of activities within the scope of practice<br>Practicum involves a variety of relevant client populations and a variety of relevant practice settings.	At least 200 hours must be supervised by an accredited exercise physiologist (provisions are available for oversight supervision when there is no suitable supervisor on site).<br>Up to 160 hours can be supervised by another professional who is qualified to be undertaking the activities they are supervising.<br>All supervisors are expected to be qualified and experienced in the activities they are supervising.<br>Requirements for maximum number of students that can be supervised at one time: Up to 1:4 (external placements); up to 1:5

Allied Health Profession	How accreditation documents describe the purpose/priorities/emphasis of what work integrated learning is for the profession	Quantity/Volume of placements	Supervision requirements for placements
		There is no expectation that hours are to be evenly split across difference areas of practice.<br>All practicum hours must involve active student engagement (passive observation cannot be used towards minimum hours requirement).	(internal placements) and there must be capacity for 1-to-1 contact with each student.
Medical Radiation Practice	Safe practice is emphasised and identified in learning outcomes.<br>Students are expected to achieve relevant pre-clinical capabilities prior to a placement.<br>Provides an opportunity for students to reflect on observations of professional practice<br>Provide students with opportunities to achieve the professional capabilities	*The quality, quantity, duration and diversity of student experience during work-integrated learning in the program is sufficient to produce a graduate who has demonstrated the knowledge, skills and professional attributes to safely and competently practise across a broad range of medical radiation practice settings*. (Standard 3.12) [51] (p. 16)<br>Students' experiences must be extensive and diverse.<br>Direct patient/client encounters are necessary for students to achieve the professional capabilities.	*Health practitioners who supervise students in the program during work-integrated learning hold current registration in Australia for the clinical elements they supervise* (Standard 1.4) [51] (p. 6)<br>For work-integrated learning in Australia, the supervisor must hold registration in the relevant division of medical radiation practice.<br>Supervision can be direct, indirect or remote.

Allied Health Profession	How accreditation documents describe the purpose/priorities/emphasis of what work integrated learning is for the profession	Quantity/Volume of placements	Supervision requirements for placements
Occupational Therapy*	Safe practice is emphasised and expected to be in the guiding principles of clinical training student learning outcomes.<br>Students are expected to achieve the relevant knowledge, skills and behaviours before interacting with the public or providing services.<br>Essential component of the curriculum.<br>Enable students to develop capacity to provide an occupational therapy service and gain registration on completion of program.<br>Provides opportunities for students to integrate knowledge, skills, and attitudes to practice with a range of people who have different needs, and in different circumstances.<br>Provides an opportunity to integrate knowledge, professional reasoning and professional behaviour with practice.<br>Provides students with an opportunity to develop the knowledge, skills and attitudes required of qualifying occupational therapists.	*Quality, quantity, duration and diversity of practice education experiences sufficient to ensure graduates are competent to practice across a range of client groups and environments reflective of current and emerging contexts.* (Standard 3.6) [53] (p. 7)<br>Students will complete sufficient practice education experiences to ensure integration of theory with practice and achieve the required competencies (WFOT [56] expectation).<br>Minimum of 1000 hours (WFOT [56] International benchmark).	*Competent and sufficient supervision is provided for students by occupational therapists and other suitably qualified supervisors to enable students to practise safely* (Standard 1.4) [53] (p. 6)<br>Supervision is provided directly or indirectly by a registered occupational therapist.<br>Direct supervision may be provided by other suitably qualified professional located on site.<br>Supervisors must have the requisite professional and supervisory skills to supervise students in the setting they are located.<br>The amount of supervision provided to students is progressively reduced as they progress towards becoming independent practitioners.

Allied Health Profession	How accreditation documents describe the purpose/priorities/emphasis of what work integrated learning is for the profession	Quantity/Volume of placements	Supervision requirements for placements
	Includes curriculum content and is an educational method.<br>Provides experiences that are consistent with the philosophy and purpose of the program.		Level of supervision will vary depending on student's learning needs and the local context.<br>Supervisors are responsible for quality of student's practice and safety of the recipient of OT (WFOT [56] expectation).<br>Innovative models are respected and valued (WFOT [56] expectation).
Optometry	Safe practice is emphasised.<br>Students are expected to achieve relevant learning outcomes before providing patient care.<br>Provides students with an extensive and diverse clinical experience across a range of settings and with a diverse range of patients and clinical presentations. Direct patient encounters provide students with this exposure and also permit development of procedural skills to achieve competency.<br>Placements provide opportunities to integrate theory into practice, experience the	*The quality, quantity and diversity of clinical training are sufficient to produce a graduate competent to practice across a range of settings and patient presentations.* (Standard 4.3) [58] (p. 14)<br>Students' clinical experiences must be extensive and diverse.<br>Direct patient encounters are necessary for students to achieve competency.<br>The range of clinical experiences must ensure graduates are safe to practise.	Supervisors should be *suitably qualified and experienced registered optometrists and/or other health professionals during clinical training*. (Standard 1.3) [58] (p. 14)<br>Supervisors must have the requisite professional and supervisory skills for student supervision.<br>Supervision can be direct or indirect.

Allied Health Profession	How accreditation documents describe the purpose/priorities/emphasis of what work integrated learning is for the profession	Quantity/Volume of placements	Supervision requirements for placements
	practice environment and build knowledge, skills and attributes essential for professional practice.	Clinical experiences should permit student development from observer to independent patient manager.<br>The guidance notes for Standard 4 indicate that a *significant period, equal to at least one equivalent full time academic year is spent primarily in direct contact with patents*.[58] (p. 15)	
Orthotics & Prosthetics	Clinical placements are integrated into the curriculum.<br>Clinical placements allow achievement of competencies across a range of practice options, client presentations and environments relevant to the Australian context.	Clinical placements allow *achievement of competencies across a range of practice areas relevant to the Australian context.* (Standard 4.5.1) [60] (p. 14)	Supervisors are *appropriately qualified as demonstrated by being appropriately credentialled to provide clinical services, and meeting the minimum AOPA competency standards* (Standard 4.5.3) [60] (p. 15)
Osteopathy	Safe practice is emphasised.<br>Placements provide opportunities to develop professional capabilities.	There must be a *broad range of student professional placements for developing graduates to meet the Osteo BA's Capabilities for osteopathic practice (2019)*. (Standard 3.9) [63] (p. 13)	Supervisors must be *registered, appropriately qualified osteopaths and/or other health practitioners* (Standard 1.6) [63] (p. 14)<br>Ratio of students to supervisor needs to be appropriate.

Allied Health Profession	How accreditation documents describe the purpose/priorities/emphasis of what work integrated learning is for the profession	Quantity/Volume of placements	Supervision requirements for placements
			Supervision can be direct or indirect.
Paramedicine	Safe practice is emphasised and identified in learning outcomes for clinical placements.<br>Students are expected to achieve relevant prerequisite capabilities before each clinical placement.<br>Provides an opportunity to integrate theory with its practical application in a professional environment.<br>Clinical placements enable students to achieve the knowledge, skills and attributes required for safe and competent practice.<br>Provides an opportunity for students to reflect on observations of professional practice.	*The quality, quantity, duration and diversity of student experience during clinical placements in the program is sufficient to produce graduates who have shown the knowledge, skills and professional attributes to safely and competently practise across a broad range of paramedicine settings*. (Standard 3.13) [66] (p. 18)<br>A minimum number of placement hours is not specified.	Supervisors must *hold current registration in Australia for the clinical elements they supervise, if practising in a regulated profession.* (Standard 1.4) [66] (p. 6)
Physiotherapy	Safe practice is emphasised.<br>Students are expected to achieve the relevant competencies before clinical placements	*The quality and quantity of clinical education is sufficient to produce a graduate competent to practise across the lifespan in a range of environments and settings*. (Standard 3.3) [66] (p. 5)	*Students are supervised by suitably qualified and registered physiotherapy and health practitioners during clinical education*. (Standard 1.4) [66] (p. 5)

Allied Health Profession	How accreditation documents describe the purpose/priorities/emphasis of what work integrated learning is for the profession	Quantity/Volume of placements	Supervision requirements for placements
	Provides opportunity for students to develop, consolidate and expand their knowledge and skills a range of areas that are key to physiotherapy practice	*The clinical placement program should provide students with experiences across acute, rehabilitation and community practice in a range of environments and settings across the lifespan.* (Australian Physiotherapy Council, 2021 [71])	
Podiatry	Safe practice is emphasised and identified in learning outcomes.<br>Students are expected to achieve relevant capabilities prior to a placement.<br>Provides an opportunity to integrate academic learning (theory) with its application to practice<br>Provides an opportunity for students to reflect on observations of professional practice.<br>Provides opportunities for students to achieve the professional capabilities.	*The quality, quantity, duration and diversity of student experience during work-integrated learning in the program is sufficient to produce a graduate who has demonstrated the knowledge, skills and professional attributes to safely and competently practise across a broad range of podiatry practice settings. This includes using pharmaceutical products for the treatment of podiatric conditions*. (Standard 3.11) [72] (p. 25)<br><br>Experiences are expected to be in a range of settings with patients from a range of age groups and clinical presentations, including patients who	*Health practitioners who supervise students during work-integrated learning hold current registration in Australia for the clinical elements they supervise* (Standard 1.4) [72] (p. 11)<br>Supervision can be by a podiatrist or another health practitioner.<br>Supervision can also be provided by health professionals who are not registered under the National Law.<br>Supervision can be direct, indirect or remote.

Allied Health Profession	How accreditation documents describe the purpose/priorities/emphasis of what work integrated learning is for the profession	Quantity/Volume of placements	Supervision requirements for placements
		are at high risk, have a range of comorbidities, are at risk of adverse outcomes related to polypharmacy and present with a range of complexities of foot and ankle pathology.	
Rehabilitation Counselling	Provides opportunity for the student to undertake appropriate activities of a Rehabilitation Counsellor under supervision	*A minimum of 200 hours field education in any year, which includes a minimum of 80 hours in direct service provision and direct client contact.*	A minimum of one hour of individual supervision each week of placement.
Social Work	Enables the integration of social work knowledge, skills and values with practice.<br>Enables the integration of classroom learning with professional practice.<br>Enables deep learning via application of knowledge, reflection, feedback and critique of experiences.<br>Socialises students into the profession<br>Enables students to build a professional identity, integrity and practice frameworks.	Minimum of 1000 hours across no more than three placements with none shorter than 250 hours.<br>At least two of the placements require distinctly different experiences.<br>At least 500 hours must be undertaken in Australia.<br>Of the 1000 hours, 500 hours must be in a direct practice role.<br>At least one placement must involve an onsite social work field educator.	Field educators must be qualified social workers with 2+ FTE years of practice experience. Must be eligible for membership with AASW.<br>There must be 1.5 hours of formal structured supervision for each 35 hours of placement. At least half of this must be one-to-one.

Allied Health Profession	How accreditation documents describe the purpose/priorities/emphasis of what work integrated learning is for the profession	Quantity/Volume of placements	Supervision requirements for placements
	Provides students with an opportunity to demonstrate the ability to meet the profession's practice standards and that they are safe, ethical and competent to practice.	All placements require active student engagement in social work roles.<br>Seven hours per 250 hours (up to a maximum of 28 hours) of practice-theory integration learning activities may be included to meet the required hours.<br>(Standards 3.1 and 3.3) [77] (p. 3)	
Speech Pathology	Students are supported on a developmental trajectory. Practice education provides opportunity for students to progressively develop competency and meet graduate competency expectations.<br>Provides opportunity for experiences across the lifespan, in a range of contexts and with a range of populations relevant to speech pathology.<br>Provides opportunity for students to integrate theory and practice.<br>Practice education program is based on a pedagogical framework	*Majority of placements must be in Australia, with service users who reside in Australia, with practice educators who reside in Australia, and assessed by practice educators who hold or are eligible for Certified Practising Speech Pathologist status.* (Criterion 32) [78] (p.15)<br>*There must be at least on near-entry level or entry-level placement in Australia which includes in -person, face-to-face service delivery*. (Criterion 32) [78] (p. 15)<br>*The majority of placements are with real, rather than simulated, service users*. (Criterion 32)[78(p15)]	The majority of placements must be *assessed by practice educators who hold or are eligible for Certified Practising Speech Pathologist status.* (Criterion 32) [78] (p. 15)

Allied Health Profession	How accreditation documents describe the purpose/priorities/emphasis of what work integrated learning is for the profession	Quantity/Volume of placements	Supervision requirements for placements
		Practice education experiences are with *individuals and communities across the lifespan in a range of contexts and with a range of populations*. (Criterion 33) [78] (p. 15)	

*The World Federation of Occupational Therapists (WFOT) is the overarching international organisation that establishes minimum standards for the education of occupational therapists. The Occupational Therapy Council of Australia draws on these for the development of the Australian accreditation standards.

Supplementary Table S2. Performance assessment on placement

(NB: Italicised text represents text extracted exactly as presented in their respective source documents.)

Allied Health Profession	Is reference made to a specific assessment tool?	Assessment on placement	Who assesses students' performance on placement?
Chinese Medicine	No	Valid and reliable assessment is emphasised. Student capability is assessed through direct observation in the clinical setting. (Standard 5.2) [37] (p. 26)	Work-integrated learning supervisors. Assessment is part of the supervision role.
Chiropractic	No	Valid assessment is emphasised Assessment of student performance through direct observation in the clinical setting is expected. (Standard 5.3) [39] (p. 8)	Not specified for placement, however it is expected that *suitably qualified and experienced staff assess students, with external input in the final year* (Standard 5.6)[39] (p. 8)
Dietetics	No	Although not explicit, it is implied across several standards that placements are where students demonstrate achievement of the National Competency Standards and that summative assessment of dietetic-specific skills against the standards is undertaken on placement.	Accredited Practising Dietitians (APD) take responsibility for summative assessment of dietetic-specific skills.

Allied Health Profession	Is reference made to a specific assessment tool?	Assessment on placement	Who assesses students' performance on placement?
Exercise Physiology	No	An assessment of performance is not stipulated although is implied as part of providing evidence for the endpoint of placements ("*competency in exercise assessment and prescription and delivery*".[48] (p. 3))<br>The accreditation documents refer to using a record of student engagement form (or equivalent) to record evidence of student's completion of practicum. This form includes a section where the placement supervisor comments on the student's performance.	There is no information on who assesses student performance on placement. However, it is stated that it is the placement supervisor who provides a comment on the record of student engagement form.
Medical Radiation Practice	No	Valid and reliable assessment is emphasised. Assessment of student capability through direct observation in the clinical setting is expected. (Standard 5.2) [51] (p. 22))<br>At least three different assessment tools or modalities are used during clinical placements to show how students meet the professional capabilities.	Work-integrated learning supervisors. Assessment is part of the supervision role.

Allied Health Profession	Is reference made to a specific assessment tool?	Assessment on placement	Who assesses students' performance on placement?
Occupational Therapy	SPEF-R2 is used widely (but not compulsory).	Fair, valid and reliable assessment is emphasised. Assessment of student performance that includes direct observation in practice education settings is expected. *Assessment strategies are diverse and varied according to the practice experience and setting. For example: 1. Observation of student performance; 2. Evaluation of student competency on SPEF-R2, 3. Practice with simulated and standardized pts, 4. Detailed client scenarios covering all of OT Process through to development of plan, 5. Student reflective diaries and assignments, 6. Learning contracts, 7. Individual and group reports.* Standard 5 Guidance notes)[54] (p. 23)	Students in the practice education setting are assessed directly or indirectly by registered occupational therapist, others suitably qualified service providers can contribute to student assessment.
Optometry	No	Fair, valid and reliable assessment is emphasised. Assessment of student performance that includes direct observation in clinical settings is expected. (Standard 6.4) [58] (p. 20)	Although not explicitly stated for clinical placements, it is expected that *suitably qualified and experienced staff, including external experts for the final year, assess students.* (Standard 6.7) [58] (p. 20)<br>Assessment is part of the clinical supervision role.

Allied Health Profession	Is reference made to a specific assessment tool?	Assessment on placement	Who assesses students' performance on placement?
Orthotics & Prosthetics	No	*Clinical placement assessment is robust and linked to learning outcomes*. (Standard 4.5.5) [60] (p. 16)	Clinical placement supervisors
Osteopathy	No	Valid assessment is emphasised.<br>Assessment of student performance through direct observation in the clinical setting is expected. (Standard 5.3) [63] (p. 14)	Osteopaths who are the placement supervisors assess students during their placements.
Paramedicine	No	Valid and reliable assessment is emphasised.<br>Assessment of student capability through direct observation in the practice setting is expected. (Standard 5.2) [66] (p. 23)<br>At least three different assessment tools or modalities are used during clinical placements to show how students meet the professional capabilities.	Work-integrated learning supervisors.<br>Assessment is part of the supervision role.
Physiotherapy	No*	Fair, valid and reliable assessment is emphasised.<br>Assessment of student performance that includes direct observation in clinical settings is expected. (Standard 5.3) [68] (p. 6)	Suitably qualified and experienced physiotherapists

Allied Health Profession	Is reference made to a specific assessment tool?	Assessment on placement	Who assesses students' performance on placement?
Podiatry	No	Valid and reliable assessment is emphasised. Assessment of student capability through direct observation in the practice setting is expected. (Standard 5.2) [72] (p. 32)<br>A range of assessment tools or modalities are used during work-integrated learning placements to show how students meet the professional capabilities.	Work-integrated learning supervisors. Assessment is part of the supervision role.
Rehabilitation Counselling	No	No information provided.	No information provided
Social Work	No	Assessment during field education should ensure students are safe, ethical and competent to practice. (Field Education Standard 3.2) [77] (p. 3)<br>*Contemporary, evidence-informed assessment activities are used, including direct observation of the student.* (Field Education Standard 3.2.2) [77] (p. 3)<br>In their final placements, students are assessed at a level that is appropriate for entry into the profession.	Field educators

Allied Health Profession	Is reference made to a specific assessment tool?	Assessment on placement	Who assesses students' performance on placement?
Speech Pathology	COMPASS® is used widely (but not compulsory)	*Assessment of students during practice education experiences in all contexts throughout the program is robust, standardised across the cohort, and linked to learning outcomes. Assessment criteria are transparent and universally applied across the cohort. (Criterion 30)* [78] (p. 15)<br>Student performance in practice education placements is assessed using COMPASS® or equivalent. *(Criterion 31)* [78] (p. 15)	Practice educators

* The APP is used widely to assess student competency, but is not referred to in the reviewed documents
